# Emerging trends in pharmacogenomics: from common variant associations toward comprehensive genomic profiling

**DOI:** 10.1186/s40246-023-00554-9

**Published:** 2023-11-24

**Authors:** Magnus Ingelman-Sundberg, Daniel W. Nebert, Volker M. Lauschke

**Affiliations:** 1 Department of Physiology and Pharmacology, Karolinska Institutet, 171 77 Stockholm, Sweden; 2 Department of Environmental and Public Health Sciences, Center for Environmental Genetics, University of Cincinnati College of Medicine, Cincinnati, OH USA; 3 Department of Pediatrics and Molecular & Developmental Biology, Cincinnati Children’s Research Center, Cincinnati, OH 45229 USA; 4 Dr Margarete Fischer-Bosch Institute of Clinical Pharmacology, Stuttgart, Germany; 5 University of Tübingen, Tübingen, Germany

## Historical perspective and origins of pharmacogenomics

Some scientists say that the “dawn of pharmacogenetics” began in southern Italy, about 510 BC, when Pythagoras described a serious illness (prevalent in some families but not others) caused by “eating fava beans”; we now know this was hemolytic anemia caused by glucose-6-phosphate dehydrogenase (G6PD) deficiency, an X-linked recessive trait that affects approximately 5% of the global population [1]. More than two millennia after this initial observation, an autosomal recessive trait of “inability to taste phenylthiourea” was reported in 1931 [2]. In 1957, Arno Motulsky was the first to propose that inheritance might explain many individual differences in the efficacy of drugs as well as occurrence of adverse drug reactions [3]. The term pharmacogenetics was coined by Friedrich Vogel in 1959 who defined it as “the study of the role of genetics in drug response” [4]. These pioneering beginnings were followed by discoveries of the autosomal recessive *N*-acetylation (*NAT2*) polymorphism [5], the autosomal dominant *ALDH2* deficiency, predominantly found in East Asians [6], and the autosomal recessive debrisoquine/sparteine oxidation (*CYP2D6*) polymorphism [7, 8]. Note that all these single Mendelian differences were characterized only by enzyme activities.

The transition from “enzyme activity pharmacogenetics” to “pharmacogenomic differences” arguably occurred in the late 1980s, when cloning, sequencing and recombinant expression of variants in *CYP2D6* [9] and *NAT2* [10] provided molecular explanations for interindividual differences in the metabolism and detoxication of numerous drugs and foreign chemicals. Since then, the Human Genome Project (which began 1 Oct 1990), and accompanying advances in sequencing technologies, have enabled the systematic profiling of pharmacogenomic variability at the population scale, which has allowed for identification of numerous gene-drug associations, and which have resulted in pharmacogenetic labels and guidelines for over 100 drugs. Since the identification of the first pharmacogenetic variants more than 30 years ago, more than 69,000 distinct single nucleotide variants (SNVs) and > 200 structural variations (SVs) have been identified across more than 200 pharmacogenes [11, 12]. Importantly, the functional relevance of the vast majority of these variants however is poorly understood, and their characterization remains an important frontier of contemporary pharmacogenomics.

## Current impact of the accumulated findings on clinical care

Personalized drug therapy encompasses the consideration of both germline and somatic genomic variations. Notably, substantial advancements have been achieved in tailoring cancer treatments through personalized pharmacotherapy. This progress primarily stems from the meticulous examination of specific mutations in growth receptors and genes pivotal to the associated signal transduction pathways. Within this context, somatic mutations in ten distinct pharmacogenes (*ALK*, *ABL1*, *BCR*, *BRCA1*, *BRAF*, *EGFR*, *ERBB2*, *KIT*, *KRAS* and *NRAS*) hold great significance in forecasting the effectiveness of cancer therapy [13, 14]. Additionally, germline variants in seven key genes (*ABCB1*, *CYP2D6*, *DPYD*, *NUDT15*, *MTHFR*, *TPMT*, and *TYMS*) play a critical role for the pharmacokinetics of anticancer medications.

In various therapeutic areas, it is evident that a limited set of genes—comprising *CYP2C9*, *CYP2C19*, *CYP2D6*, *CYP3A5*, *DPYD*, *TPMT*, *SLCO1B1*, and *HLA-B*—took precedence in clinical trials conducted between 2012 and 2020, with a specific focus on specific gene-drug pair interactions [15]. Remarkably, these same genes, in addition to *NAT2* and *UGT1A1*, have now assumed significance within the pharmacogenomic domain according to the FDA, with the data supporting therapeutic management recommendations [16]. When it comes to polymorphic genes impacting pharmacokinetics, *CYP2C19* and *CYP2D6* stand out as the predominant genes to consider [17]. It is thus worth noting that the repertoire of polymorphic genes with clinical relevance has remained small in number and relatively stable in recent years.

Within different therapeutic areas, the most significant impact of preemptive genotyping historically has been observed in the field of oncology, which has also considered germline mutations. Within other areas, interestingly, 66% of the 50 distinct drugs are pertinent to the central nervous system (mainly epilepsy, psychosis, depression and neurology) and 8% are associated with cardiovascular disease, leaving a mere four drugs within other therapeutic areas [16]. Presently, the utility of preventive pharmacogenetic testing in psychiatry remains an open question, but it undeniably demands substantial attention in the forthcoming years.

## Confounding factors in pharmacogenomic research

The successful identification of gene-drug associations requires the application of appropriate pharmacogenomic assays in well-characterized patient cohorts of sufficient size to power statistically meaningful conclusions. However, many pharmacogenomic studies have been conducted in heterogeneous patient populations lacking careful phenotypic stratification and do not sufficiently consider environmental and pathophysiological factors, such as patients’ liver and kidney function. Broader clinical trials in this realm face substantial challenges stemming from various confounding factors. Specifically, a critical concern revolves around closed-label studies, making it exceedingly difficult to blind physicians to the treatment conditions. The impact of placebo effects within randomized controlled trials (RCTs) is substantial, as exemplified in the case of treating mental depression with new selective serotonin reuptake inhibitors (SSRIs), where placebos can contribute to as much as 50% of the observed effects [18]. Polypharmacy and resulting drug-drug interactions (DDIs), is another noteworthy variable—especially among older patients, who often require in excess of five concomitant medications to address their health conditions [19].

For purposes of comparison, studies should be designed to encompass a substantial proportion of patients possessing functional variants pertinent to each drug under scrutiny in the context of the particular therapeutic intervention. Another challenge pertains to the elucidation of heritability, as the genetic basis of up to 50% of hereditary variability in drug pharmacokinetics remains unknown [20]. Consideration should also be given to employ hard quantitative endpoints, such as differences in drug concentrations in pharmacokinetic assessments because classifications of response or adverse events can be more subjective. The challenges mentioned above were manifest in the recent PREPARE trial, in which patients receiving standard treatment displayed a comparable reduction in adverse effects to those undergoing genotype-guided drug therapy [21, 22].

## Future aspects

Impactful improvements of pharmacogenomics analyses can be expected in different areas. From a technical standpoint, the development of long-read sequencing technologies offers exciting prospects to improve the identification of genetic variation. Long-read sequencing technologies have already been shown to provide accurate variant calls and to facilitate the reliable phasing of diplotypes of complex pharmacogenomic genes, such as *CYP2D6* or the locus harboring the *HLA* genes encoding the major histocompatibility complex [23, 24]. The recent maturation of this technology from targeted profiling of a few candidate genes to the comprehensive and rapid sequencing of complete fully phased human genomes [25, 26] opens exciting opportunities to advance the field from variant to haplotype associations also in other genomic loci.

In addition, phasing information can also provide useful information for the generation of polygenic risk scores (PRSs), which are being increasingly adopted in pharmacogenomics [27]. Currently, PRSs constitute an aggregation of many individual polymorphisms, sometimes tens of thousands [28], and are calculated as the weighted sum of the effect sizes of all incorporated variants. As a result, complex genetic signatures can be collapsed into a single score, which correlates with the genetic predisposition for the given trait. PRSs have been successfully used for the stratification of patients into ezetimibe responders and non-responders [29], for the prediction of lurasidone response in schizophrenia [30], and for the identification of heart failure patients who benefit most from beta-blocker therapy [31]. To further extend the utility of PRSs, it will be important to develop theoretical frameworks that allow to extend associations from single variants to haplotypes.

To accompany the rapid profiling of genomic sequence, methods are required that aid in the functional interpretation of the identified variation. To this end, major leaps have been made in the development of pharmacogenetic “variant effect” predictors. Many commonly used computational algorithms have been developed to identify pathogenic variations and, thus, are focused on variations with known disease associations [32]. This approach, however, does not yield accurate predictions when applied to pharmacogenetic variations, which, while often deleterious, are mostly not under considerable evolutionary constraints. Consequently, interpretation of variants in such poorly conserved pharmacogenes requires the use of dedicated, specialized algorithms [33]. Despite their clear improvement in performance, compared to prior tools, further optimizations will be required to make computational algorithms fit for routine clinical utilization in pharmacogenomics. Specifically, moving forward, it will be important to further improve their calibration using large-scale training data sets, e.g., generated via deep mutational scanning [34, 35]. Further, it will be important to see whether emerging AI-based structural prediction tools, such as AlphaMissense [36], can provide reliable variant effect predictions for pharmacogenetic variations.

## Implementation into the clinics

Given the complexities illustrated above, the clinical integration of pharmacogenomics presents a formidable challenge. The foremost instrument facilitating translation is the incorporation of pharmacogenomic data within drug information labels. Nonetheless, the availability of these labels is lacking in numerous countries, and their utilization in clinical settings remains sporadic. Hence, it becomes imperative for policymakers, healthcare practitioners, patients, and regulatory authorities to champion the adoption of preemptive genotyping as a means to enhance patient healthcare. A compelling rationale for such implementation resides in the realm of sound pharmacoeconomic investigations, which furnish compelling arguments in favor of the cost–benefit of testing. A current meta-analysis [37] found that among the included 108 studies pertaining to 39 pharmaceutical agents, 71% were cost-effective or cost-saving. Nevertheless, there remains a lack of pivotal research endeavors capable of convincing regulatory decision-makers.

In addition to the cost-effectiveness of a given test, it is also important to consider the cost-effectiveness of required infrastructural investments to enable timely and reliable genotype analyses at the national level. This infrastructure should ideally encompass centralized laboratories endowed with state-of-the-art equipment and expertise. It is of great importance to educate both healthcare providers about the background and potential use of pharmacogenomics. We also need to make sure this knowledge is shared with patient advocacy groups. Anticipation surrounds the notion that widespread implementation will be expedited when the merits of pharmacogenomics within healthcare are more persuasively demonstrated through the identification of an expanded array of genetic variants and the outcomes of rigorously designed, blinded, randomized clinical trials.

## Conclusions

Considering the *holistic* nature of each unique human being, it is perhaps not surprising that each individual’s response to virtually every drug would be extremely complex. Shaping these differences is a combination of genetics and epigenetic effects, endogenous influences, environmental exposures, and each individual’s microbiome. These factors can further be interdependent. Among the heritable variability one can classify (i) monogenic (Mendelian) traits, typically influenced by one or a few variants, (ii) oligogenic traits that usually represent variability largely elicited by a small number of major pharmacogenes and (iii) complex pharmacogenomic traits, produced by innumerable small-effect variants [38]. Note that, except for germline variability, the other categories are not constant, but rather are continuously changing throughout one’s lifetime [39]. These concepts would explain why even monozygotic twins might exhibit pharmacogenomic differences in drug response.

The field of pharmacogenomics has undergone substantial advancements in the past decade; however, the current progress has yet to yield widely dependable clinical tools that are adopted on a significant scale. In essence, we find ourselves at a stage that marks not the beginning of the end but rather the end of the beginning in the development of this field. A recent study, utilizing a GWAS in an extensive cohort of 5.4 million subjects, revealed a noteworthy correlation between height and 12,111 independent SNVs in 7209 non-overlapping genomic segments, covering approximately 21% of the genome [40]. While one can imagine that the genetics of height is likely more intricate than that of drug response, achieving similar saturation of drug response heritability appears to be a monumental task. Encouragingly, there is rapid progress in sequencing methods, but validating these genetic variants demand extensive and costly clinical trials, often hindered by inadequate funding.

With diminishing costs and increasing accessibility of technology to everyone, it has been suggested that complete genome assemblies, in which both parental haplotypes are phased, telomere to telomere, will become standard in human genetics [41]. We believe that complete genetic variant discovery will transform how we map, catalog, and associate variations—not only with human diseases, but also with responses to drugs.

Immediate actions however are hampered by a scarcity of high-quality phenotype data, essential for robust genotype–phenotype correlations. Absence of such data poses a significant obstacle to enhancing our understanding of how genetic variations impact drug responses. Our focus should thus strategically shift toward conducting meticulous studies that scrutinize specific gene-drug pairs. By directing our attention to these targeted investigations, we are poised to gain crucial insights with profound implications for clinical applications.
